# Supplementary material for: GDF-15 Inhibits ADP-Induced Human Platelet Aggregation through the GFRAL/RET Signaling Complex
Source: Biomolecules. 2023 Dec 27;14(1):38. doi: 10.3390/biom14010038 (PMC10813690; doi:10.3390/biom14010038)

Figure S1 Human platelets do not express TGF-βRII and HER2

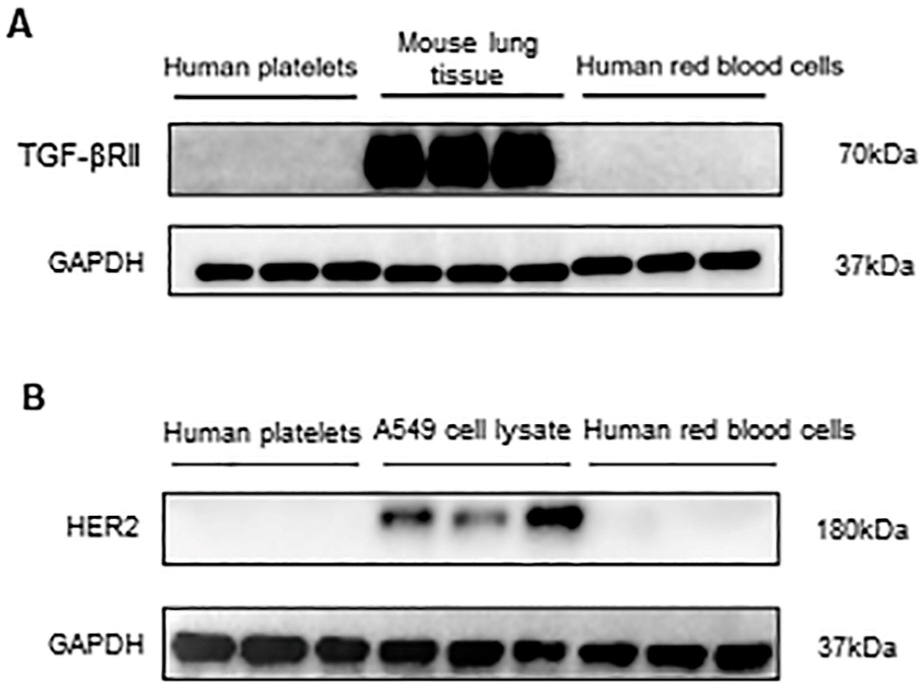

Original western blots images of Figure S1

Protein name: TGF-βRII Predicted band size: 70kDa

The sequence for the samples is as follow (from the left): human platelets 1, human platelets 2, human platelets 3, mouse lung tissue 1, mouse lung tissue 2, mouse lung tissue 3, human red blood cells 1, human red blood cells 2, human red blood cells 3.

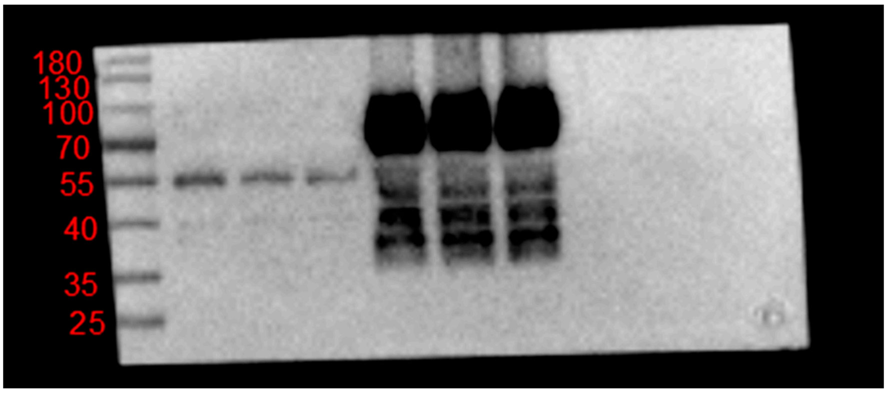

Protein name: GAPDH Predicted band size: 37kDa

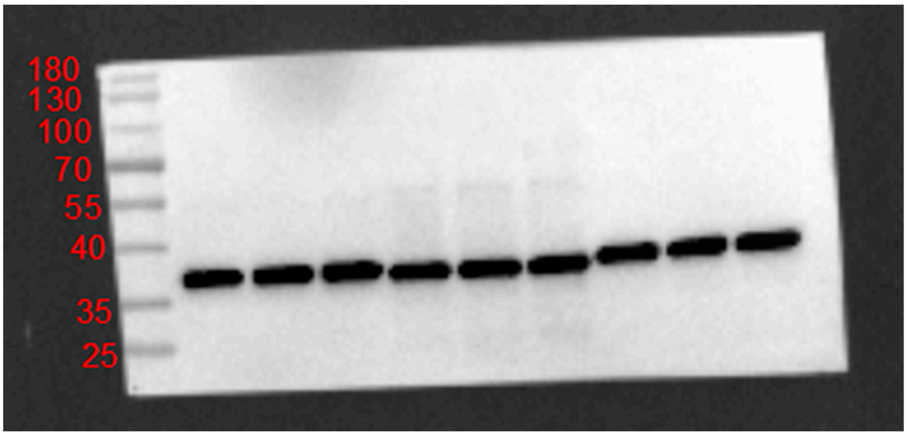

Protein name: **HER2** Predicted band size: **180kDa**

The sequence for the samples is as follow (from the left): human platelets 1, human platelets 2, human platelets 3, A549 cell lysate 1, A549 cell lysate 2, A549 cell lysate 3, human red blood cells 1, human red blood cells 2, human red blood cells 3.

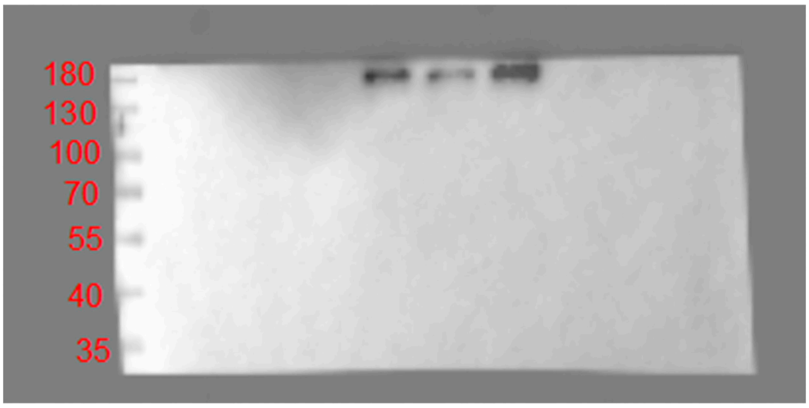

Protein name: GAPDH Predicted band size: 37kDa

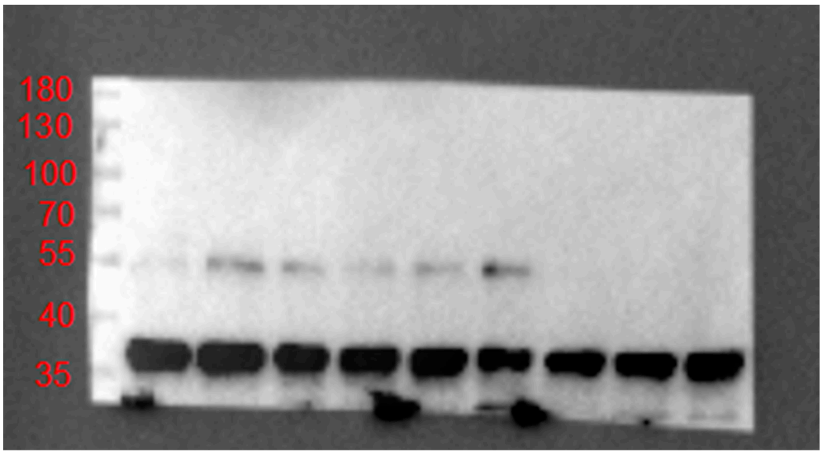

Original western blots images of Figure 2A-C

Protein name: **GFRAL**　　　Observed band size:**52kDa**

1.The sequence for the samples is as follow (from the left):

platelets①, platelets②, platelets③, red blood cells①, red blood cells②, red blood cells ③, white blood cells①, 　white blood cells②, white blood cells③

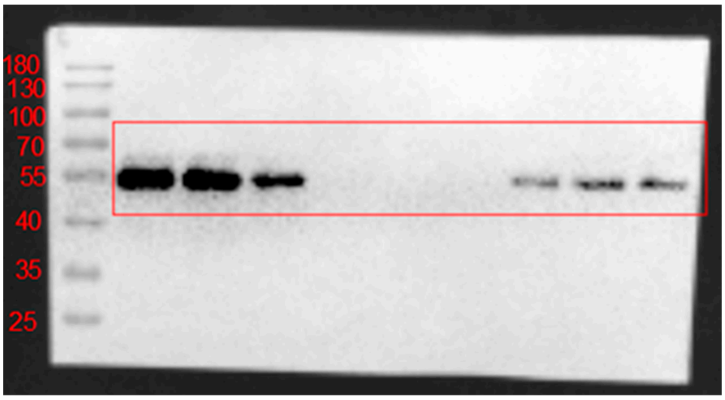

2. The sequence for the samples is as follow (from the left):

platelets④, platelets⑤, platelets⑥,red blood cells④, red blood cells⑤, red blood cells ⑥,white blood cells④, white blood cells⑤, white blood cells⑥.

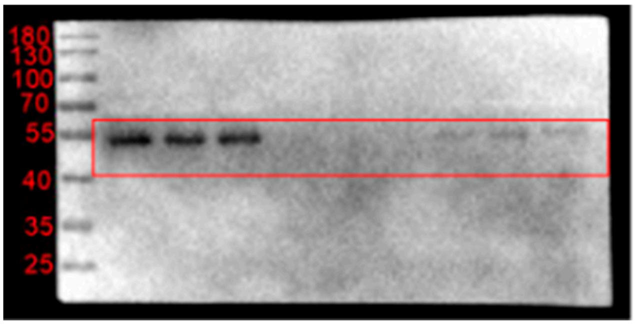

Protein name: **RET**　　　　　Observed band size:**180kDa**

1.The sequence for the samples is as follow (from the left):

platelets①, platelets②, platelets③,red blood cells①, red blood cells②, red blood cells ③,white blood cells①, white blood cells②, white blood cells③

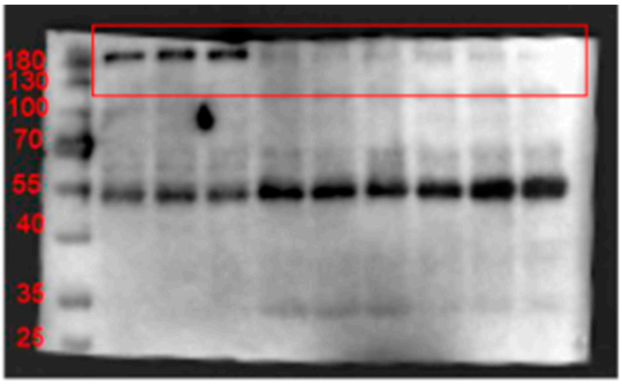

2.The sequence for the samples is as follow (from the left):
platelets④, platelets⑤, platelets⑥,red blood cells④, red blood cells⑤, red blood cells ⑥,white blood cells④, white blood cells⑤, white blood cells⑥.

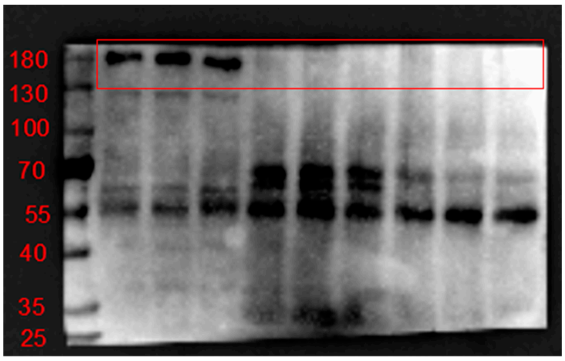

Protein name: **GAPDH** Observed band size:**37kDa**

1.The sequence for the samples is as follow (from the left):
platelets①, platelets②, platelets③,red blood cells①, red blood cells②, red blood cells ③,white blood cells①, white blood cells②, white blood cells③

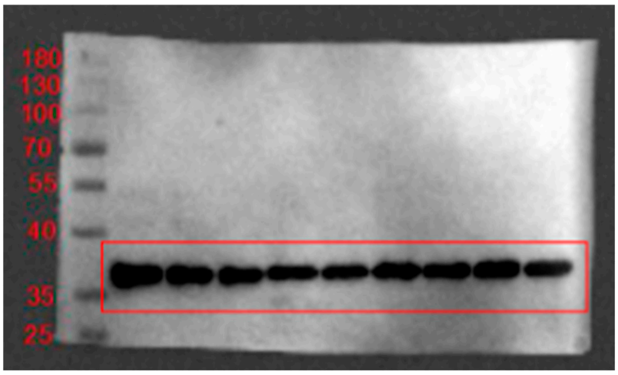

2.The sequence for the samples is as follow (from the left):
platelets④, platelets⑤, platelets⑥,red blood cells④, red blood cells⑤, red blood cells ⑥,white blood cells④, white blood cells⑤, white blood cells⑥.

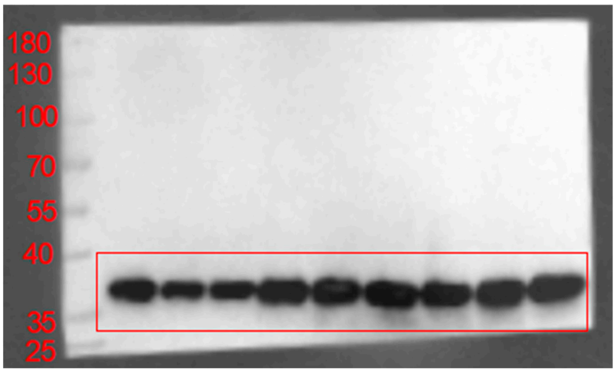

Protein name: **TGF-βRI** Observed band size:**55kDa**

1.The sequence for the samples is as follow (from the left):
platelets①, platelets②, platelets③,red blood cells①, red blood cells②, red blood cells ③,white blood cells①, white blood cells②, white blood cells③

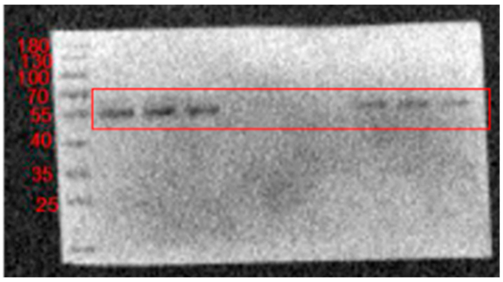

2.The sequence for the samples is as follow (from the left):
platelets④, platelets⑤, platelets⑥,red blood cells④, red blood cells⑤, red blood cells ⑥,white blood cells④, white blood cells⑤, white blood cells⑥.

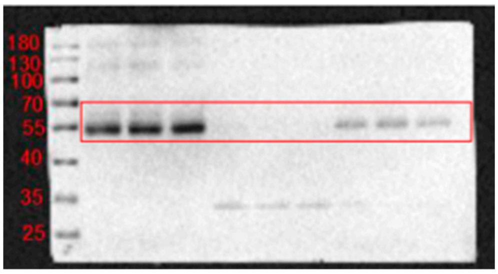

Protein name: **TGF-βRII** Predicted band size: **70kDa**

1.The sequence for the samples is as follow (from the left):
platelets①, platelets②, platelets③,red blood cells①, red blood cells②, red blood cells ③,white blood cells①, white blood cells②, white blood cells③

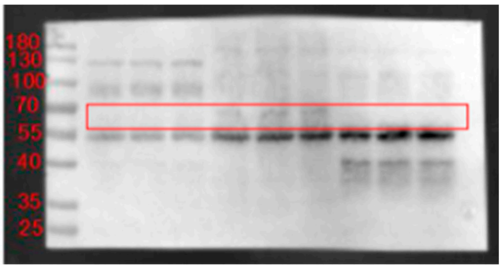

2.The sequence for the samples is as follow (from the left):
platelets④, platelets⑤, platelets⑥,red blood cells④, red blood cells⑤, red blood cells ⑥,white blood cells④, white blood cells⑤, white blood cells⑥.

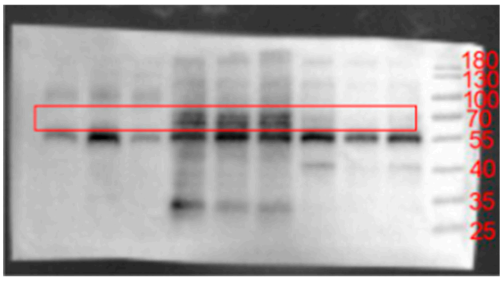

Protein name: **GAPDH** Observed band size:**37kDa**

1.The sequence for the samples is as follow (from the left):
platelets①, platelets②, platelets③,red blood cells①, red blood cells②, red blood cells ③,white blood cells①, white blood cells②, white blood cells③

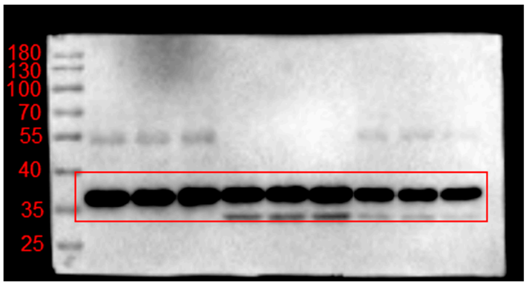

2.The sequence for the samples is as follow (from the left):
platelets④, platelets⑤, platelets⑥,red blood cells④, red blood cells⑤, red blood cells ⑥,white blood cells④, white blood cells⑤, white blood cells⑥.

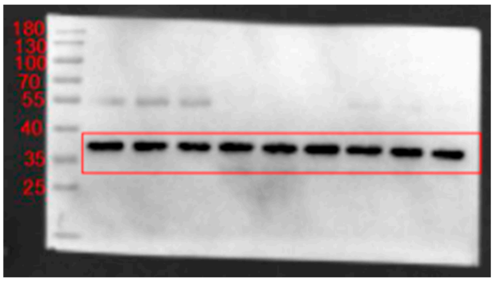

Protein name: **EGFR** Observed band size:**130kDa**

1.The sequence for the samples is as follow (from the left):
platelets①, platelets②, platelets③,red blood cells①, red blood cells②, red blood cells ③,white blood cells①, white blood cells②, white blood cells③

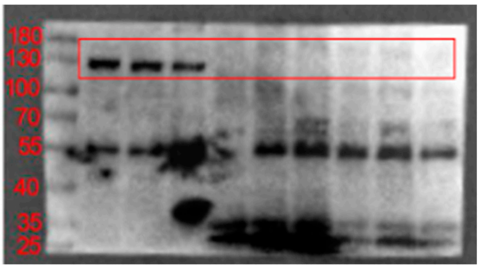

2.The sequence for the samples is as follow (from the left):
platelets④, platelets⑤, platelets⑥,red blood cells④, red blood cells⑤, red blood cells ⑥,white blood cells④, white blood cells⑤, white blood cells⑥.

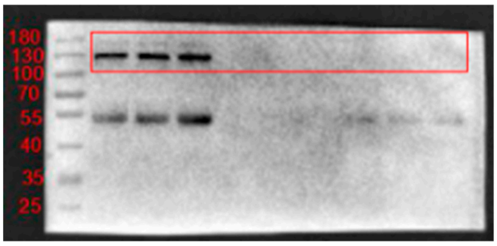

Protein name: **HER2** Predicted band size:**180kDa**

1.The sequence for the samples is as follow (from the left):
platelets①, platelets②, platelets③,red blood cells①, red blood cells②, red blood cells ③,white blood cells①, white blood cells②, white blood cells③

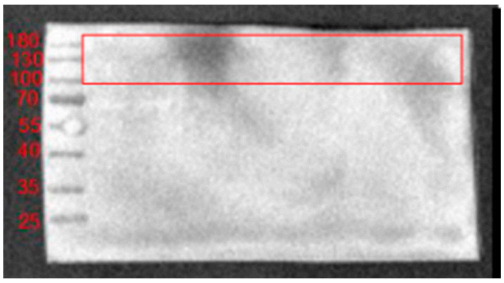

2.The sequence for the samples is as follow (from the left):
platelets⑤, platelets⑤, platelets⑥,red blood cells④, red blood cells⑤, red blood cells ⑥,white blood cells④, white blood cells⑤, white blood cells⑥.

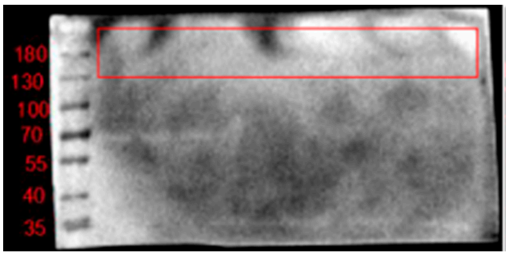

Protein name: **HER3** Observed band size:**150-210kDa**

1.The sequence for the samples is as follow (from the left):
platelets①, platelets②, platelets③,red blood cells①, red blood cells②, red blood cells ③,white blood cells①, white blood cells②, white blood cells③

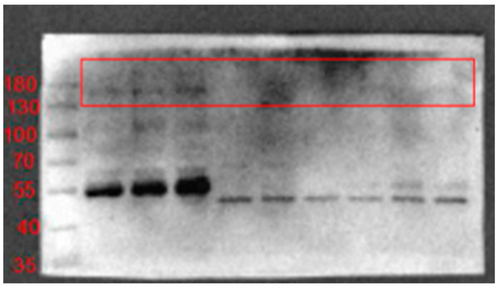

2.The sequence for the samples is as follow (from the left):
platelets④, platelets⑤, platelets⑥,red blood cells④, red blood cells⑤, red blood cells ⑥,white blood cells④, white blood cells⑤, white blood cells⑥.

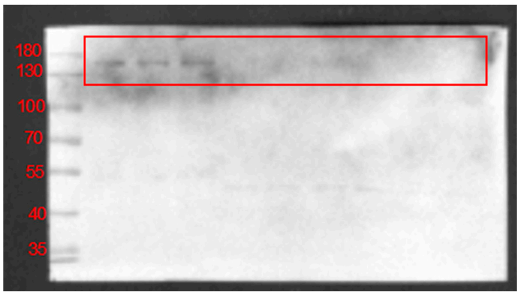

Protein name: **GAPDH** Observed band size:**37kDa**

1.The sequence for the samples is as follow (from the left):
platelets①, platelets②, platelets③,red blood cells①, red blood cells②, red blood cells

③,white blood cells①, white blood cells②, white blood cells③

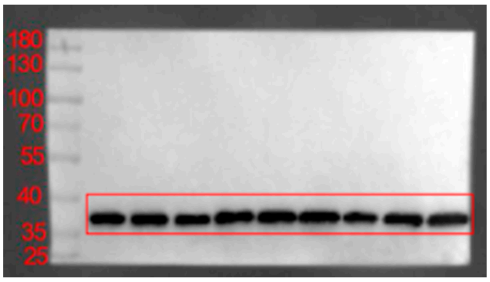

2.The sequence for the samples is as follow(from the left):
platelets④, platelets⑤, platelets⑥,red blood cells④, red blood cells⑤, red blood cells ⑥,white blood cells④, white blood cells⑤, white blood cells⑥.

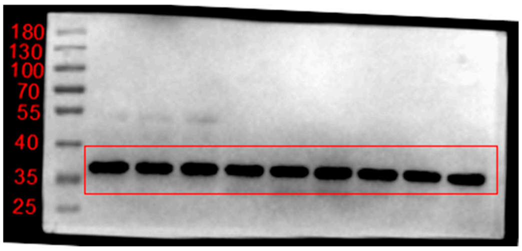

Original western blots images of Figure 3B

Protein name: **GDF-15** Observed band size:**13kDa**

1. The sequence for the samples is showed in the images (a duplicate well is set up for each sample):

Repeat 1 Repeat 2 Repeat 3

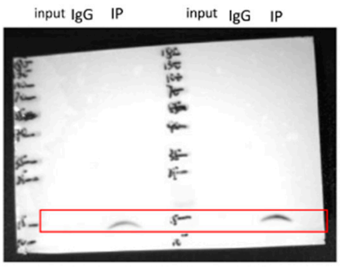

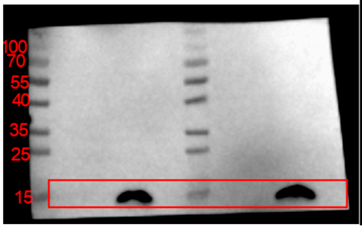

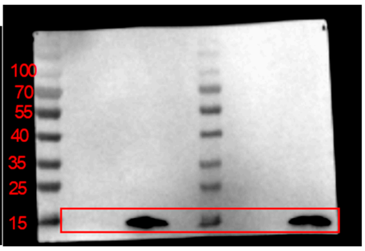

Protein name: **GFRAL** Observed band size:**53kDa**

The sequence for the samples is showed in the images (a duplicate well is set up for each sample):

Repeat 1 Repeat 2 Repeat 3

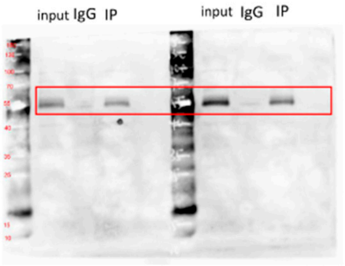

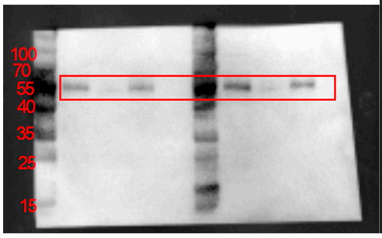

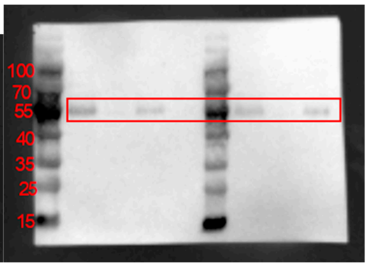

Original western blots images of Figure 4A-C

Protein name: **phospho-ERK** Observed band size:**42,44kDa**

1.The sequence for the samples is as follow (from the left):

CTRL①, CTRL②, CTRL③,ADP①, ADP②, ADP③,ADP+GDF-15①, ADP+GDF-15②, ADP+GDF-15③

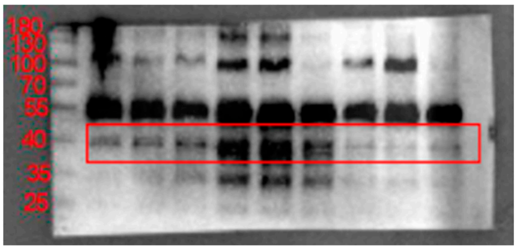

2.The sequence for the samples is as follow (from the left):

CTRL④, CTRL⑤, CTRL⑥,ADP④, ADP⑤, ADP⑥,ADP+GDF-15④, ADP+GDF-15⑤, ADP+GDF-15⑥

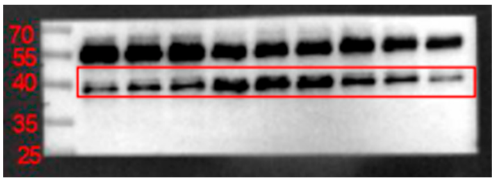

Protein name: **total-ERK** Observed band size:**42,44kDa**

1.The sequence for the samples is as follow (from the left):

CTRL①, CTRL②, CTRL③,ADP①, ADP②, ADP③,ADP+GDF-15①, ADP+GDF-15②, ADP+GDF-15③

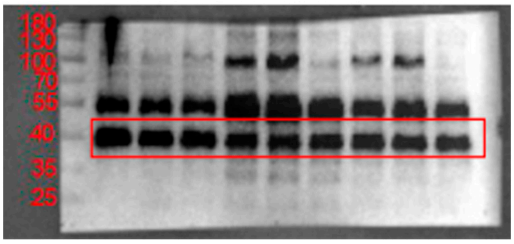

2.The sequence for the samples is as follow (from the left):

CTRL④, CTRL⑤, CTRL⑥,ADP④, ADP⑤, ADP⑥,ADP+GDF-15④, ADP+GDF-15⑤, ADP+GDF-15⑥

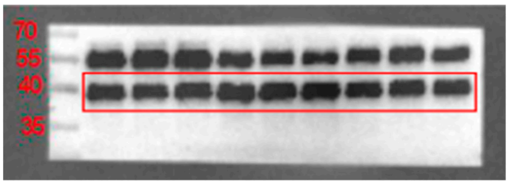

Protein name: **GAPDH** Observed band size:**37kDa**

1.The sequence for the samples is as follow (from the left):

CTRL①, CTRL②, CTRL③,ADP①, ADP②, ADP③,ADP+GDF-15①, ADP+GDF-15②, ADP+GDF-15③

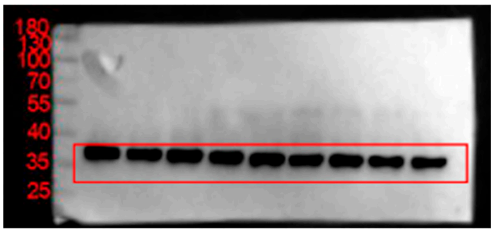

2.The sequence for the samples is as follow (from the left):
CTRL④, CTRL⑤, CTRL⑥,ADP④, ADP⑤, ADP⑥,ADP+GDF-15④, ADP+GDF-15⑤, ADP+GDF-15⑥

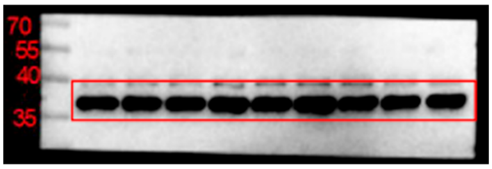

Protein name: **phospho-AKT** Observed band size:**60kDa**

1.The sequence for the samples is as follow (from the left):
CTRL①, CTRL②, CTRL③,ADP①, ADP②, ADP③,ADP+GDF-15①, ADP+GDF-15②, ADP+GDF-15③

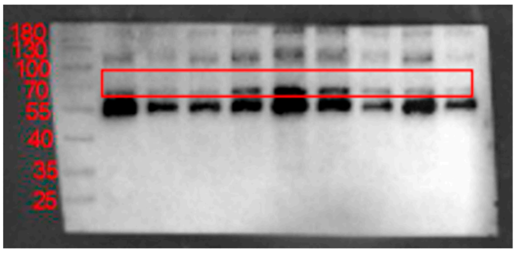

2.The sequence for the samples is as follow (from the left):
CTRL④, CTRL⑤, CTRL⑥,ADP④, ADP⑤, ADP⑥,ADP+GDF-15④, ADP+GDF-15⑤, ADP+GDF-15⑥

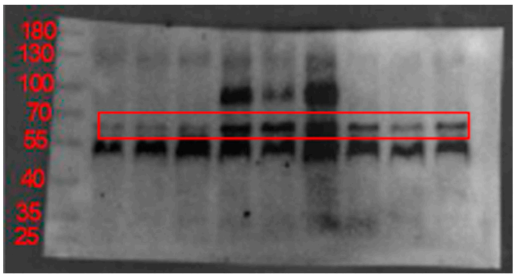

Protein name: **total-AKT** Observed band size:**60kDa**

1.The sequence for the samples is as follow (from the left):
CTRL①, CTRL②, CTRL③,ADP①, ADP②, ADP③,ADP+GDF-15①, ADP+GDF-15②, ADP+GDF-15③

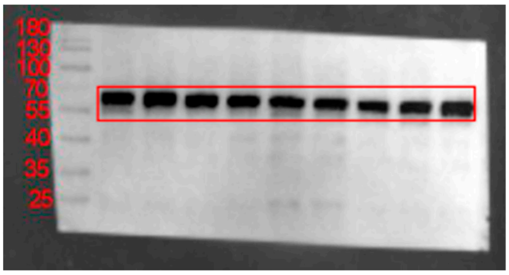

2.The sequence for the samples is as follow (from the left):
CTRL④, CTRL⑤, CTRL⑥,ADP④, ADP⑤, ADP⑥,ADP+GDF-15④, ADP+GDF-15⑤, ADP+GDF-15⑥

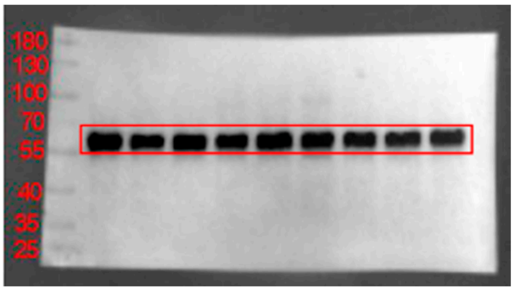

Protein name: **GAPDH** Observed band size:**37kDa**

1.The sequence for the samples is as follow (from the left):
CTRL①, CTRL②, CTRL③,ADP①, ADP②, ADP③,ADP+GDF-15①, ADP+GDF-15②, ADP+GDF-15③

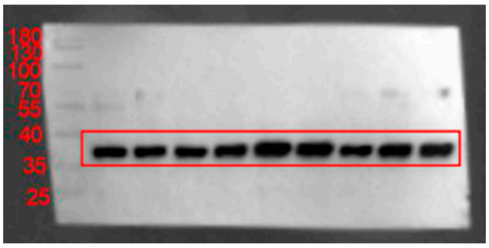

2.The sequence for the samples is as follow (from the left):
CTRL④, CTRL⑤, CTRL⑥,ADP④, ADP⑤, ADP⑥,ADP+GDF-15④, ADP+GDF-15⑤, ADP+GDF-15⑥

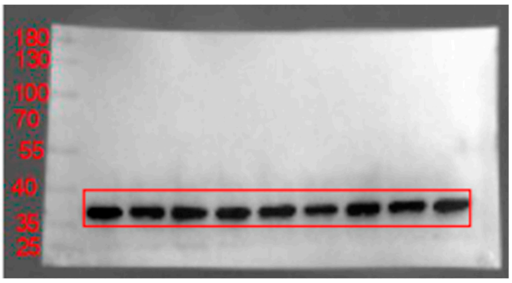

Protein name: **phospho-JAK2** Observed band size:**125kDa**

1.The sequence for the samples is as follow (from the left):
CTRL①, CTRL②, CTRL③,ADP①, ADP②, ADP③,ADP+GDF-15①, ADP+GDF-15②, ADP+GDF-15③

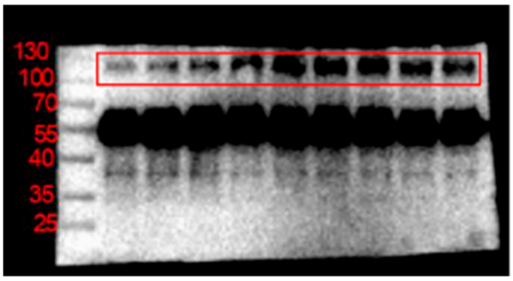

2.The sequence for the samples is as follow (from the left):
CTRL④, CTRL⑤, CTRL⑥,ADP④, ADP⑤, ADP⑥,ADP+GDF-15④, ADP+GDF-

15⑤, ADP+GDF-15⑥

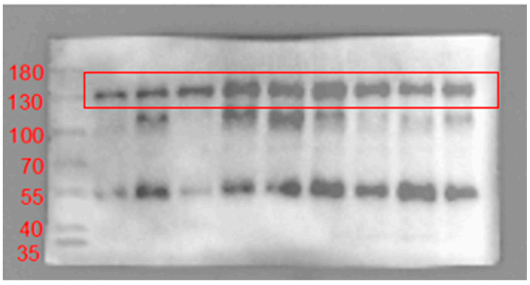

Protein name: **total-JAK2** Observed band size:**130kDa**

1.The sequence for the samples is as follow (from the left):

CTRL①, CTRL②, CTRL③,ADP①, ADP②, ADP③,ADP+GDF-15①, ADP+GDF-15②, ADP+GDF-15③

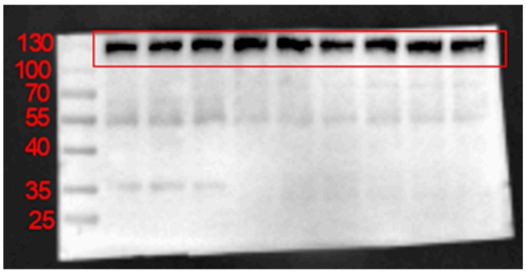

2.The sequence for the samples is as follow (from the left):

CTRL④, CTRL⑤, CTRL⑥,ADP④, ADP⑤, ADP⑥,ADP+GDF-15④, ADP+GDF-15⑤, ADP+GDF-15⑥

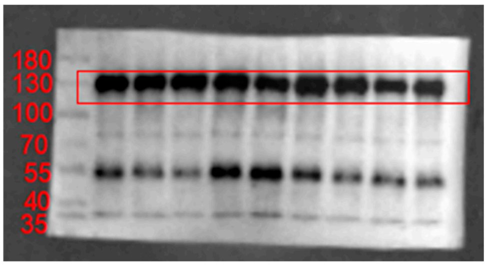

Protein name: **GAPDH** Observed band size:**37kDa**

1.The sequence for the samples is as follow (from the left):

CTRL①, CTRL②, CTRL③,ADP①, ADP②, ADP③,ADP+GDF-15①, ADP+GDF-15②, ADP+GDF-15③

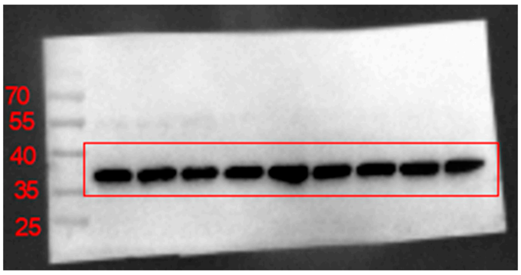

2.The sequence for the samples is as follow (from the left):

CTRL④, CTRL⑤, CTRL⑥,ADP④, ADP⑤, ADP⑥,ADP+GDF-15④, ADP+GDF-15⑤, ADP+GDF-15⑥

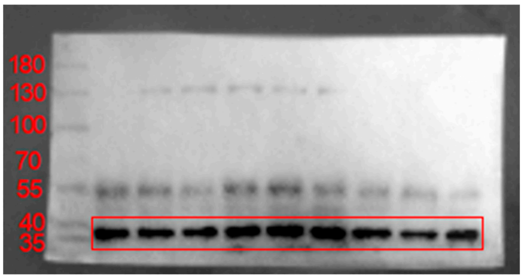

Original western blots images of Figure 5A

一、AKT pathway

1.Protein name: **phospho-AKT** Observed band size:**60kDa**

The sequence for the samples is as follow (from the left):

CTRL, ADP, ADP+GDF-15, ADP+GDF-15+BT-13, ADP+GDF-15+SPP-86

①

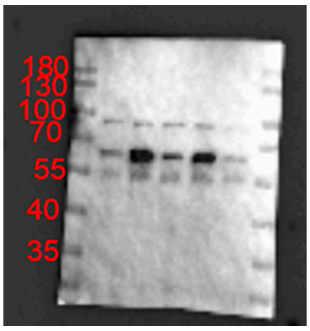

②

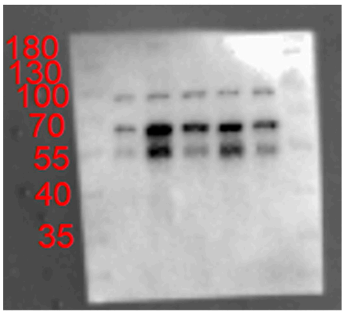

③

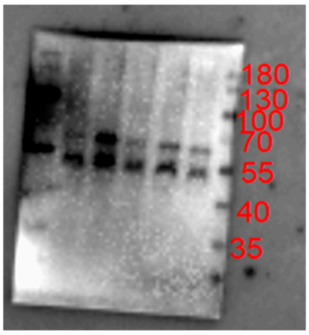

④

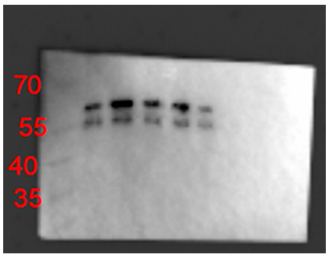

⑤

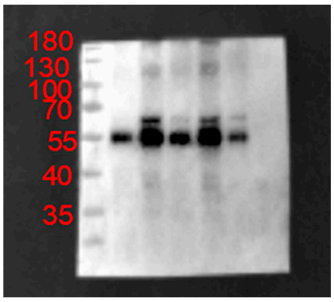

⑥

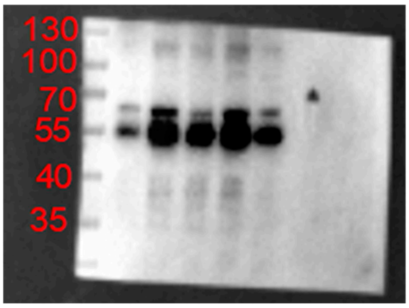

2.Protein name: **total-AKT** Observed band size:**60kDa**

The sequence for the samples is as follow (from the left):

CTRL, ADP, ADP+GDF-15, ADP+GDF-15+BT-13, ADP+GDF-15+SPP-86

①

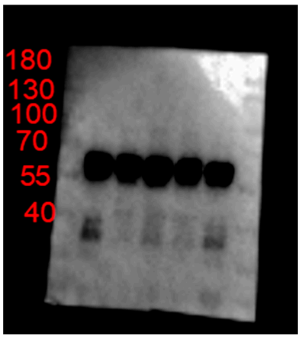

②

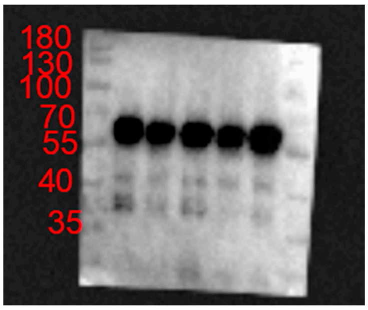

③

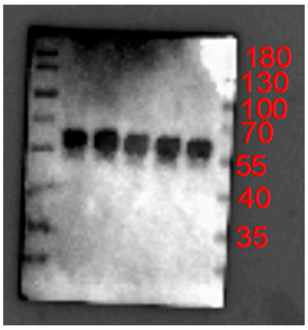

④

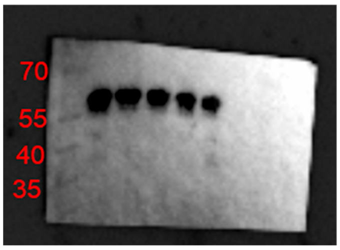

⑤

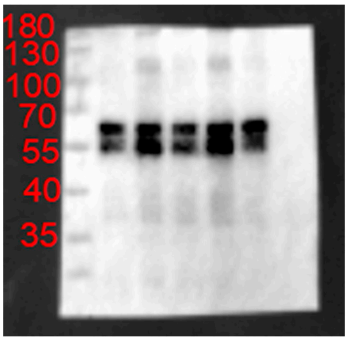

⑥

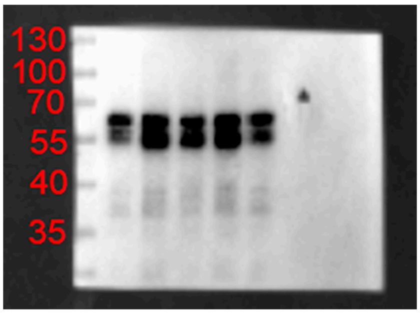

3.Protein name: **GAPDH** Observed band size:**37kDa**

The sequence for the samples is as follow (from the left):

CTRL, ADP, ADP+GDF-15, ADP+GDF-15+BT-13, ADP+GDF-15+SPP-86

①

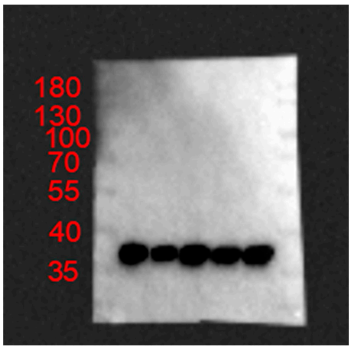

②

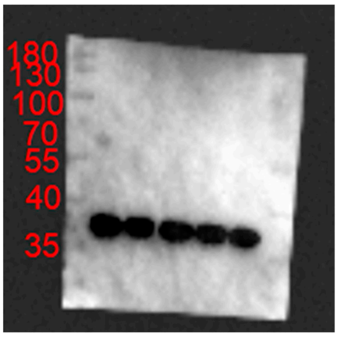

③

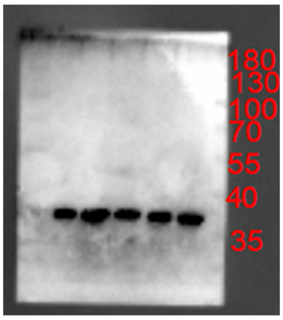

④

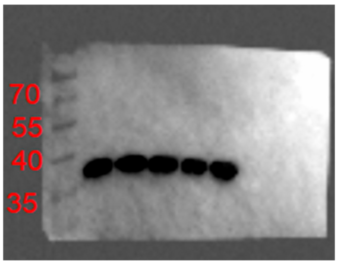

⑤

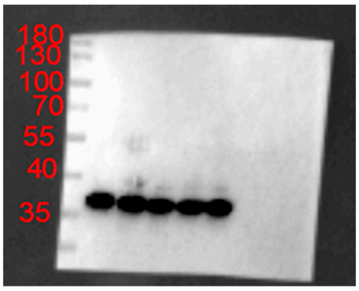

⑥

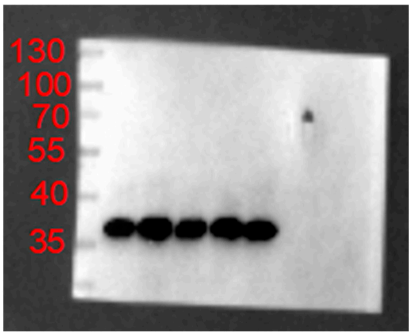

二、ERK pathway

1.Protein name: **phospho-ERK** Observed band size:**42/44kDa**

The sequence for the samples is as follow (from the left):

CTRL, ADP, ADP+GDF-15, ADP+GDF-15+BT-13, ADP+GDF-15+SPP-86

①

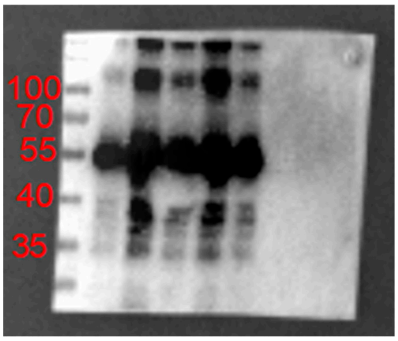

②

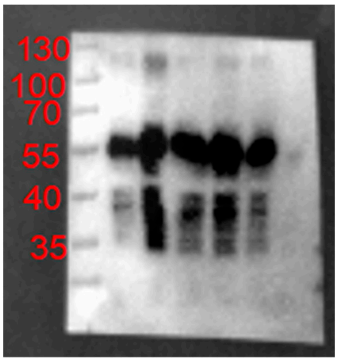

③

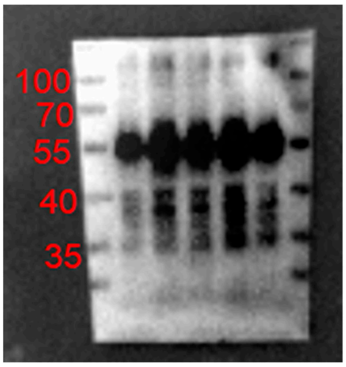

④

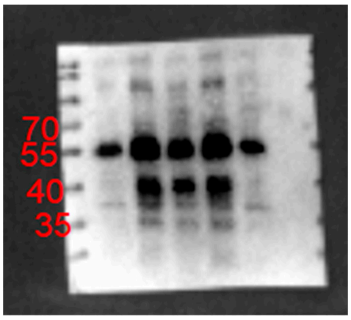

⑤

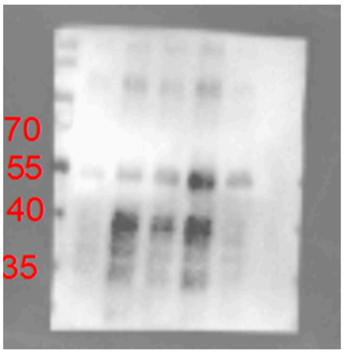

⑥

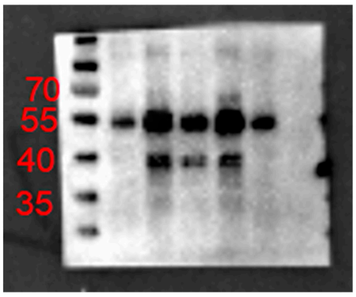

2.Protein name: **total-ERK** Observed band size:**42/44kDa**

The sequence for the samples is as follow (from the left):

CTRL, ADP, ADP+GDF-15, ADP+GDF-15+BT-13, ADP+GDF-15+SPP-86

① ②

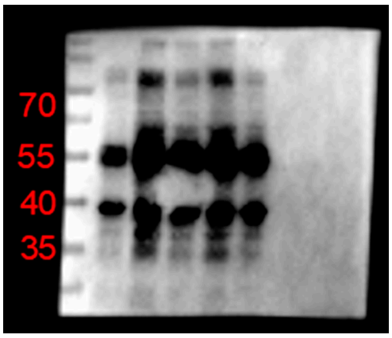

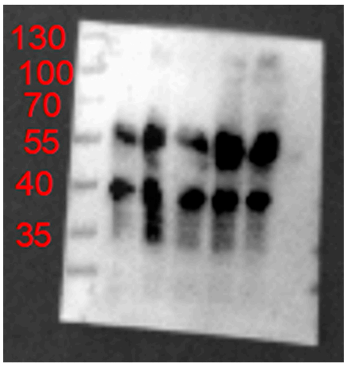

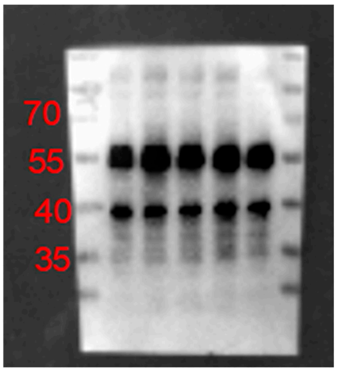

④ ⑤ ⑥

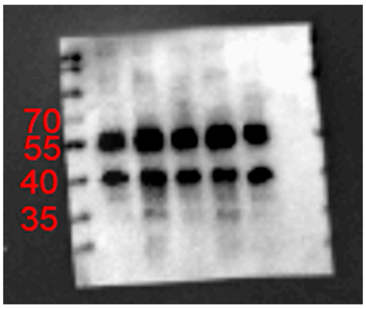

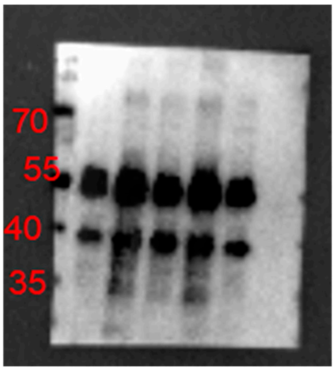

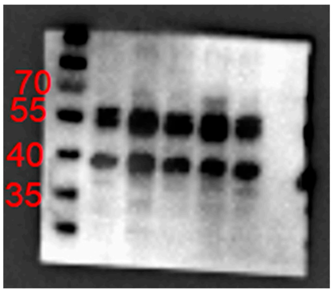

3.Protein name: **GAPDH** Observed band size:**37kDa**

The sequence for the samples is as follow (from the left):

CTRL, ADP, ADP+GDF-15, ADP+GDF-15+BT-13, ADP+GDF-15+SPP-86

① ② ③

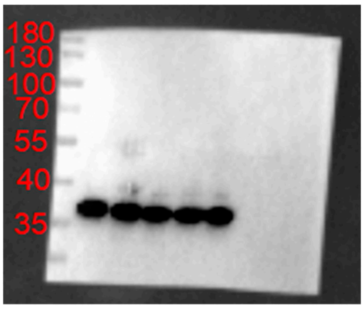

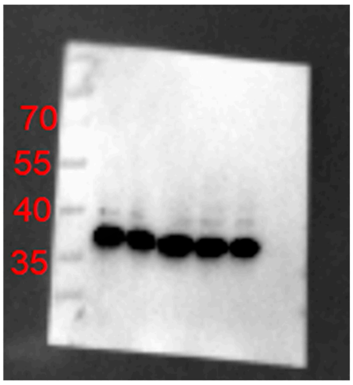

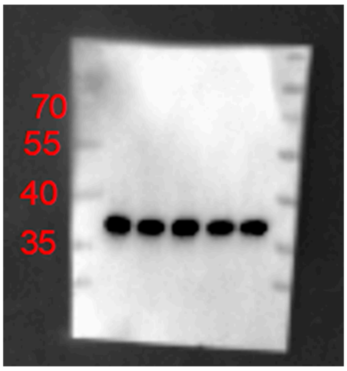

④

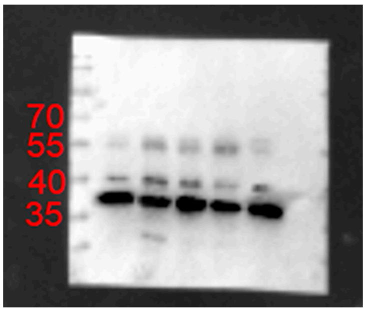

⑤

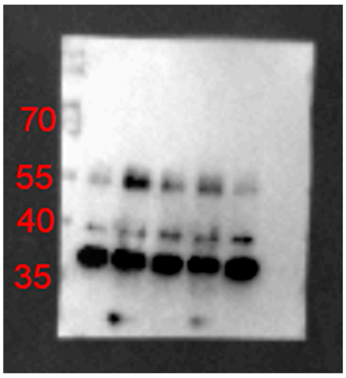

⑥

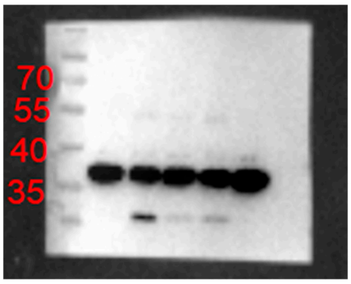

Supplement: Supplementary file 1 [file biomolecules-14-00038-s001.zip › biomolecules-2723732-supplementary.pdf]
